# Efficacy of neurofeedback-assisted mindfulness for anxiety and nomophobia in Spanish young adults: a randomized controlled trial

**DOI:** 10.3389/fpubh.2025.1712564

**Published:** 2025-12-02

**Authors:** Mei Fernandez-Crespo, M. Isabel Rihuete-Galve, Natalia Sanchez-Aguadero, Nerea Sanchez-Sanchez, Jesus Gonzalez-Sanchez, Irene A. Garcia-Yu, Hsin-Chien Lee, Jose I. Recio-Rodriguez

**Affiliations:** 1 Facultad de Enfermería y Fisioterapia, Universidad de Salamanca (USAL), Salamanca, Spain; 2 Instituto de Investigación Biomédica de Salamanca (IBSAL), Salamanca, Spain; 3 Unidad de Investigación de Atención Primaria de Salamanca (APISAL), Salamanca, Spain; 4 Hospital Universitario de Salamanca, Salamanca, Spain; 5 Facultad de Medicina, Universidad de Salamanca (USAL), Salamanca, Spain; 6 Research Center of Sleep Medicine, College of Medicine, Taipei Medical University, Taipei, Taiwan; 7 Red de Investigación en Cronicidad, Atención Primaria y Promoción de la Salud (RICAPPS), Barcelona, Spain

**Keywords:** neurofeedback, mindfulness, anxiety, nursing, young adults, randomized controlled trial

## Abstract

**Background:**

Problematic use of new technologies has been associated with anxiety and other disorders in young adults. This study evaluated the efficacy of a Neurofeedback-Assisted Mindfulness Training Program (NAMTP) in reducing anxiety and related symptoms in young adults.

**Methods:**

A randomized clinical trial assigned participants to an intervention group (IG), receiving 20-25 NAMTP sessions, or a control group (CG). All participants received a brief workshop on responsible technology use. Anxiety was assessed using the DASS-21 anxiety subscale. Secondary outcomes included the Smartphone Addiction Scale—Short Version (SAS-SV), Nomophobia Questionnaire (NMP-Q), and Athens Insomnia Scale (AIS), all evaluated at baseline and three months.

**Results:**

A total of 40 participants completed the study (82.5% women; mean age 24.8 ± 3.4). No statistically significant differences were observed between the intervention group (IG) and the control group (CG) in any of the psychological variables assessed. For the DASS scales, the mean differences (IG–CG) were 0.1 (95% CI: −1.6 to 1.9; *p* = 0.868) for anxiety, −2.4 (95% CI: −7.2 to 2.4; *p* = 0.324) for depression, and −1.5 (95% CI: −5.3 to 2.4; *p* = 0.453) for stress. Similarly, the mean difference in the SAS-SV score was −1.0 (95% CI: −5.0 to 2.9; *p* = 0.606), while for the NMP-Q score it was 3.7 (95% CI: −8.9 to 16.4; *p* = 0.553). Finally, the Athens Insomnia score showed a mean difference of −1.1 (95% CI: −2.9 to 0.7; *p* = 0.211). Adherence to the NAMTP program was high (88.8%).

**Conclusions:**

The NAMTP did not produce significant improvements in anxiety, mobile phone addiction, nomophobia, or sleep quality. These findings suggest that, although NAMTP is feasible and well-tolerated, further research is needed to determine its potential benefits.

**Trial registration:**

Clinicaltrials.gov; NCT06188910. http://www.Clinicaltrials.gov/ct2/show/NCT06188910.

## 1 Introduction

The use of new technologies, the internet, and mobile devices has increased in recent years, transforming the way we interact, obtain information, and entertain ourselves. Despite their benefits, the excessive and inappropriate use of these tools has led to growing concern about their negative impact on physical and mental health, especially in young people.

### 1.1 The effects of problematic technology use among young people

Various studies have linked problematic use of new technologies with symptoms of anxiety, depression, low self-esteem, eye strain (1, 2), attention deficit hyperactivity disorder (ADHD), sleep problems, hostility (3, 4), and reduced academic and work performance (5), among other issues (6). This phenomenon is especially alarming among children and adolescents, due to their developmental vulnerability and ongoing brain maturation.

Sleep is essential for healthy development, and insufficient sleep has become a public health concern. Longer screen time has been associated with poorer sleep quality, shorter sleep duration, and delayed bedtimes, especially in adolescents and young adults (7–9). While poor sleep may also lead to increased screen use, evidence suggests that evening smartphone exposure disrupts circadian rhythms and delays melatonin release (10). Notably, restricting mobile phone use before bedtime for several weeks has been shown to reduce sleep latency, improve sleep quality and duration, and enhance mood and cognitive performance (11).

A systematic review has indicated an association between problematic social media use and the presence of depression and anxiety in children under the age of 18. The mechanisms that have been identified include social comparison, sleep deprivation, and feedback-seeking behaviors (12). Several studies have demonstrated a significant positive association between problematic smartphone use (PSU) and anxiety symptom severity, consistently categorized as a small-to-moderate effect (13, 14). A comprehensive metanalysis identified that PSU significantly increases the risk of anxiety, with pooled odds ratio around 3.5 (15). From a theoretical perspective, anxiety may contribute to PSU through compensatory mechanisms, whereby excessive technological engagement serves as a maladaptive strategy for managing negative emotions. This association is supported by factors such as the Fear of Missing Out (FOMO), which mediates the link between anxiety and PSU severity (13, 14), and Negative Metacognition (NM), identified as key predictors in the pathway connecting anxiety dimensions with smartphone addiction (16).

Previous authors have suggested this excessive engagement as a behavioral addiction exhibiting addiction-like characteristics (17, 18). This problem exhibits high global prevalence. In Spain, 11.3% of individuals aged 15 and 24 who use the internet are at high risk of compulsive use (6). Globally, a systematic review revealed a 71% prevalence of internet addiction among university students in India (19). Similarly, research in Brazil found that 34.3% of participants reported problematic use, identifying female sex and screen time as key risk factors (20). Within this context, nomophobia—defined as the fear of being without a mobile phone—has become a specific focus of study. A meta-analysis examining its global prevalence across 52 studies from various continents found that moderate levels of nomophobia were the most prevalent, noting a significant upward trend in recent years (21). A predictive model of nomophobia highlighted the roles of fear of missing out, non-pathological compulsion, and rumination as key mechanisms explaining the condition (22).

### 1.2 Therapeutic approaches to technology addiction: the emerging role of mindfulness

In recent years, psychological, pharmacological, and healthy habit promotion strategies have been studied to address addiction to new technology. A meta-analysis demonstrated the efficacy of interventions such as Cognitive Behavioral Therapy (CBT), physical activity, pharmacological treatments, and group therapy in reducing internet addiction (23).

Among these, mindfulness has gained relevance due to its capacity to improve self-control and reduce symptoms such as nomophobia (24). It has been shown that even a single mindfulness session can alleviate symptoms associated with excessive mobile phone use (25), and that regular practice can be key in preventing addictive behaviors (24, 26).

Recent research supports its application. A study in adolescents showed that a mindfulness program acted as a mediator in PSU, improving levels of depression and sleep problems (27). Other research evaluated short online mindfulness programs, with daily 10-min sessions over 30 days, observing efficacy in reducing various forms of mobile addiction: mobile social media addiction, mobile game addiction, mobile in-formation acquisition addiction, and mobile short-form video addiction (28). The combined use of mindfulness and Tai Chi Chuan has also shown significant improvements in both mobile addiction and inhibition capacity, a key component in controlling addictive behaviors (29).

However, evidence regarding sleep quality is mixed. For example, in a group of nurses with insomnia, mindfulness treatment did not show significant improvements compared to the standard treatment (CTB), which proved more effective (30).

### 1.3 Neurofeedback as a complementary approach to mindfulness and addiction treatment

Mindfulness interventions are beneficial but often challenging to implement broadly, as they typically require specialized instructors and structured training. In this context, neurofeedback-assisted mindfulness emerges as a more accessible and scalable alternative. Neurofeedback (NF) is a biofeedback technique that trains individuals to self-regulate their brain activity through real-time feedback of electrical signals from the brain, mainly recorded through electroencephalography (EEG) (31, 32). This technique is valuable because it has demonstrated the ability to increase the power of alpha waves -associated with attentional focus- even in subjects with no prior meditation experience (26).

NF is a therapeutic technique aimed at the prevention, optimization, and rehabilitation of altered states of cortical activity, which has been shown to be effective in the treatment of various conditions, including anxiety, depression, ADHD, and post-traumatic stress disorder (PTSD) (33). Regarding anxiety disorders, evidence supports its effectiveness, as indicated by the clinical guidelines of the Canadian Agency for Drugs and Technologies in Health (34), which reported significant improvements in patients with PTSD and generalized anxiety compared to those who did not receive treatment (34). Similarly, a clinical trial in young university students with generalized anxiety disorder showed that the group treated with NF experienced a significant reduction in anxiety and depression levels, along with enhanced emotional regulation compared to the control group (35).

At a psychological level, research supports NF's potential in addictions. A recent meta-analysis evaluating EEG-NF effects on addiction reported symptoms improvement, especially in substance-related addictions. Behavioral addictions, such as internet addiction, showed lower heterogeneity, suggesting similar patterns of brain dysregulation (36). EEG studies in individuals with internet addiction reveal a pattern characterized by reduced beta activity and increased gamma activity (37), along with a predominance of slow frontal waves, which is associated with a deficit in inhibitory control (38). This opens a pathway for NF use to restore brain activity balance.

Based on a recent systematic review, mindfulness-based interventions assisted by NF enhance functional connectivity within and between key brain networks, improving self-regulation and cognitive control (39). This practice has been shown to influence activity in the insula (40) and the posterior cingulate cortex (41), two brain areas implicated in processes such as rumination, a central mechanism in nomophobia (22).

Initial studies on the combined application are promising. A randomized clinical trial among children and adolescents in South Korea demonstrated significant improvements on the Internet Addiction Scale following participation in a NF program guided by a mobile application (42). However, similar to the mindfulness findings, studies on NF focused on alpha waves have found non-significant results regarding objective sleep measures (43), consistent with other reviews concluding that NF offers no additional benefits for insomnia compared to other treatments (44).

### 1.4 Objectives and hypotheses

The primary objective of this study was to evaluate the efficacy of a Neurofeedback-Assisted Mindfulness Training Program (NAMTP) in reducing anxiety among young Spanish adults. The secondary objectives were to examine the program's effects on mobile phone addiction, nomophobia, and sleep quality.

The study hypothesized that the NAMTP would demonstrate a positive effect in reducing anxiety. The combination of NF and mindfulness was expected to be more effective because it uses real-time feedback to train the brain through reinforcement learning. This is a more robust way to build self-regulation skills (45), needed to manage anxiety.

## 2 Methods

### 2.1 Study design

A two-arm, parallel-group randomized clinical trial was conducted in Salamanca, Spain, from February 2024 to February 2025. This randomized controlled trial was conducted in accordance with the Consolidated Standards of Reporting Trials (CONSORT) guidelines (46).

The clinical trial was registered at clinicaltrials.gov with the code NCT06188910 on January 02, 2024.

The study protocol has been published previously (47). The main results of this study are presented in this manuscript.

### 2.2 Study participants and recruitment

Participants were recruited through social media platforms and posters displayed in local health centers that advertised the study. Volunteers from the Salamanca area who met the following inclusion criteria were recruited: aged between 18 and 35, regular smartphone users, fully capable of completing the questionnaires, and having signed the informed consent form. Exclusion criteria included a history of serious psychiatric disorders, such as bipolar disorder, major depressive episode, or other non-organic psychotic disorders, requiring treatment within the 6 months prior to enrollment; or any brain injuries or conditions that contraindicated the use of NF.

### 2.3 Sample size

The sample size was estimated based on the primary outcome variable of anxiety, as measured by the Depression, Anxiety and Stress Scale (DASS-21). According to the study by Abdian et al. (35), it was determined that a total of 40 participants were required to detect a significant difference of 3.9 points or greater. The estimation was calculated with an alpha risk of 0.05 and a beta risk of 0.2, estimating a follow-up loss of 20%.

### 2.4 Randomization and blinding

Following the baseline assessment, participants were randomly assigned in a 1:1 ratio to either the control group (CG) or the intervention group (IG). Simple randomization was performed using a computer-generated sequence created by Epidat 4.2. software.

To ensure allocation concealment, a member of the research team who was not involved in participant enrollment was responsible for the randomization process.

After the baseline assessment was completed, participants were assigned a study identification number; this information was sent to the independent researcher, who used the pre-generated concealed allocation list to determine the participant's assigned group and informed the nurse responsible for delivering the intervention.

Due to the nature of the intervention, neither the participants nor the fieldwork nurse could be blinded to group assignments.

### 2.5 Intervention

All study participants in both groups (CG and IG) received an initial 30-min educational workshop on the responsible use of mobile phones and new technologies, conducted by a researcher.

The workshop included slides and printed handouts summarizing digital balance concepts and practical advice for healthy technology use. It began with a brief introduction explaining key concepts, followed by a guided reflection on personal technology use and strategies to establish healthy boundaries, concluding with a summary emphasizing awareness of digital identity and footprint. It took place individually in a designated room at the Nursing Faculty of the University of Salamanca.

The workshop was delivered identically to all participants, with minor clarifications or examples provided upon questions but no individualized modifications. No changes were made relative to the original protocol, and adherence was recorded for all participants, ensuring that all workshop components were delivered according to the planned schedule.

In addition to this workshop, only the IG received the NAMTP, which consisted of 20–25 sessions held 2–3 times per week. Each session lasted between 10 and 15 min. The number and duration of sessions were determined based on prior literature and studies with similar methodologies, which employed short EEG-based mindfulness or neurofeedback interventions. These studies reported measurable improvements in internet addiction disorder after 20 sessions, providing a basis for selecting a similar protocol in this study to explore comparable effects (36, 48). Furthermore, this dosage aimed to balance intervention intensity with participant adherence while maintaining sufficient training exposure.

Although traditional EEG systems retain superior spatial resolution and signal fidelity compared to mobile consumer-grade devices (49), the latter's value proposition is strongly rooted in accessibility and portability. According to a recent scoping review, consumer-grade EEG devices have demonstrated considerable utility across diverse research domains, thereby reinforcing the potential of affordable, portable technology to expand access to brain research and promote a more inclusive and equitable approach to neuroscience (50).

The study used the MUSE^®^ device (InteraXon Inc.), specifically the Muse 2 and Muse S Headband models, which record EEG signals from four channels: an electrode on each side of forehead (AF7-AF8), two sensors behind the ears (TP9-TP10) and a reference electrode in the center of the forehead (FPz) (51, 52). Recognized as a cost-efficient mobile-EEG tool, the MUSE^®^ has demonstrated validated utility in capturing electrophysiological markers of basic sensory and cognitive processes (51, 53, 54). Previous MUSE^®^-based studies have established these markers as reliable behavioral correlations of attentional control and mindfulness engagement, showing reductions in mind wandering (55, 56).

Through its sensors, the device records brain signals and transmits them via Bluetooth to the *Muse: EEG Meditation & Sleep* mobile app, which uses machine learning algorithms to analyze brain waves and classify the mental state as calm, neutral, or active. The algorithm processes brain activity in the delta (1–4 Hz), theta (4–8 Hz), alpha (8–13 Hz), beta (13–30 Hz), and gamma (30–44 Hz) frequency bands to estimate the participant's mental state. Real-time auditory neurofeedback is generated (52).

Because the feedback parameters of the MUSE^®^ system are proprietary and not manually adjustable, the indicators “recoveries” and “birds” were recorded as indirect neurofeedback metrics. “Recoveries” represent the number of times the participant successfully refocused attention after distraction, while “birds” indicate sustained periods of calm (≥5 s) as detected by the algorithm.

For the initial sessions, the guided programs *Discover Mind Biofeedback* and *MUSE Essentials* were used to introduce participants to mindfulness practice. Beginning with the 17th session, participants transitioned to independent auditory-feedback programs to train self-regulation. Each session began with a 2-min introduction to a theoretical concept or practical tip by a meditation and mindfulness instructor. This was followed by a mindfulness practice with real-time NF and concluded with a 1–2-min reflection on effective tools and sensations.

IG participants attended the NAMTP sessions. Individual adjustments were made within the IG to mindfulness techniques (such as body scanning or breath counting) to optimize attention and engagement. Attendance was recorded according to the protocol, tracking each participant's session sequence to ensure fidelity. No changes were made to the original protocol.

Given that the NF device (MUSE^®^) is a non-invasive EEG system providing auditory feedback, no serious risks were anticipated. Potential adverse effects included mild skin irritation, headache or fatigue, transient drowsiness, and emotional discomfort or mild anxiety during mindfulness practice. Participants were instructed to report any new or undesirable symptoms, which were then recorded and classified by the research staff according to severity (mild, moderate, severe) and potential relation to the intervention.

The CG received no additional intervention beyond the initial educational workshop.

### 2.6 Instruments

The primary outcome, anxiety, was assessed using the *Depression, Anxiety and Stress Scale 21-item (DASS-21)*. The Spanish version evaluated emotional states via three 7-item subscales (depression, anxiety, and stress). The presence of adverse mental health symptoms was considered according to the cut-off points proposed by Roman et al. (57) in the validation study with a sample of young people in Spain: DASS-21-Depression ≥6 points, DASS-21-Anxiety ≥6 points, and DASS-21-Stress ≥5 points.

The secondary outcomes were measured using the following scales:

- *Smartphone Addiction Scale (SAS-SV):* A 10-item tool where scores > 31 indicate smartphone addiction risk. The scale, adapted to Spanish population, has shown excellent internal consistency (α = 0.88) and good construct validity, with a single-factor structure explaining 49.3% of the variance (58).
- *Nomophobia Questionnaire (NMP-Q):* It is an instrument composed of 20 items. The range of scores varies from 20 to 140 points, with higher scores indicating a greater risk and severity of nomophobia. The Spanish adaptation of the Nomophobia Questionnaire (NMP-Q) showed excellent internal consistency (ordinal α = 0.95) and good test–retest reliability (r = 0.823) (59).
- *Athens Insomnia Scale (AIS):* This is an 8-item tool that helps in the diagnosis and assessment of insomnia according to ICD-10 diagnostic criteria. Higher scores reflect worse insomnia symptoms. The Spanish adaptation showed good internal consistency (α = 0.86), acceptable test–retest reliability (ICC = 0.75) (60).

All variables were assessed at baseline and 3 months after randomization. For each outcome, the mean and standard deviation were calculated, as well as the difference between the two time points. Statistical analyses were performed to determine whether there were significant differences over time and between groups.

### 2.7 Data collection procedure and management

A trained nurse was responsible for participant recruitment, scheduling intervention sessions, and data collection at both baseline and 3-month follow-up. Data adherence was monitored through attendance records.

Each participant was assigned a unique identifier to link their records across study visits. Paper records were kept under lock and key at the faculty, while the team compiled electronic records in a private database accessible only to authorized study researchers.

### 2.8 Ethical considerations

The study was approved by the Clinical Research Ethics Committee of the Salamanca Health Area (CEIm Code: PI 2023 07 1340). All participants provided written informed consent, in accordance with the Declaration of Helsinki.

All data were handled in strict compliance with current data protection regulations, including the EU General Data Protection Regulation (GDPR 2016/679) and Spanish legislation: Organic Law 3/2018 on Personal Data Protection and Digital Rights, along with Law 14/2007 on biomedical research. Confidentiality was maintained throughout the study in accordance with these legal frameworks.

### 2.9 Statistical analysis

Statistical analysis was performed according to the study protocol using IBM SPSS Statistics for Windows, version 28.0. An intention-to-treat (ITT) analysis was performed, including all participants regardless of their adherence to the target number of sessions. In cases of missing post-intervention data, available baseline data were used for descriptive analyses, while missing outcome data were excluded from pre–post comparisons. No imputation was planned unless deemed necessary.

Quantitative variables were expressed as mean ± standard deviation, while categorical variables were expressed by number and frequency. The Kolmogorov-Smirnov test was used to test the normality of variables.

Descriptive analyses were conducted using mean difference for quantitative variables and the chi-square test for categorical variables.

The association between variables was analyzed with Student's *t*-test and analysis of variance (ANOVA). The Student's *t*-test for independent samples was used to com-pare the means between two groups, evaluating the change within the same group with the Student's *t*-test for paired data.

A repeated-measures General Linear Model (GLM) was conducted in SPSS to examine changes in anxiety, depression, stress and smartphone addiction, nomophobia and insomnia before and after the intervention, with the group as the between-subjects factor and baseline anxiety scores as a covariate. The model included a within-subjects factor with two levels (pre and post), using Type III sums of squares and a significance level of α =0.05. Profile plots with confidence intervals were generated to visualize group differences, and estimated marginal means were computed adjusted for the covariate, with *post-hoc* comparisons performed using the LSD correction. In addition, descriptive statistics, effect size (η^2^), and observed power were obtained to assess the magnitude and reliability of the findings. The statistical significance limit was set at an alpha risk of 0.05.

## 3 Results

### 3.1 Participants flow

**Figure 1:**
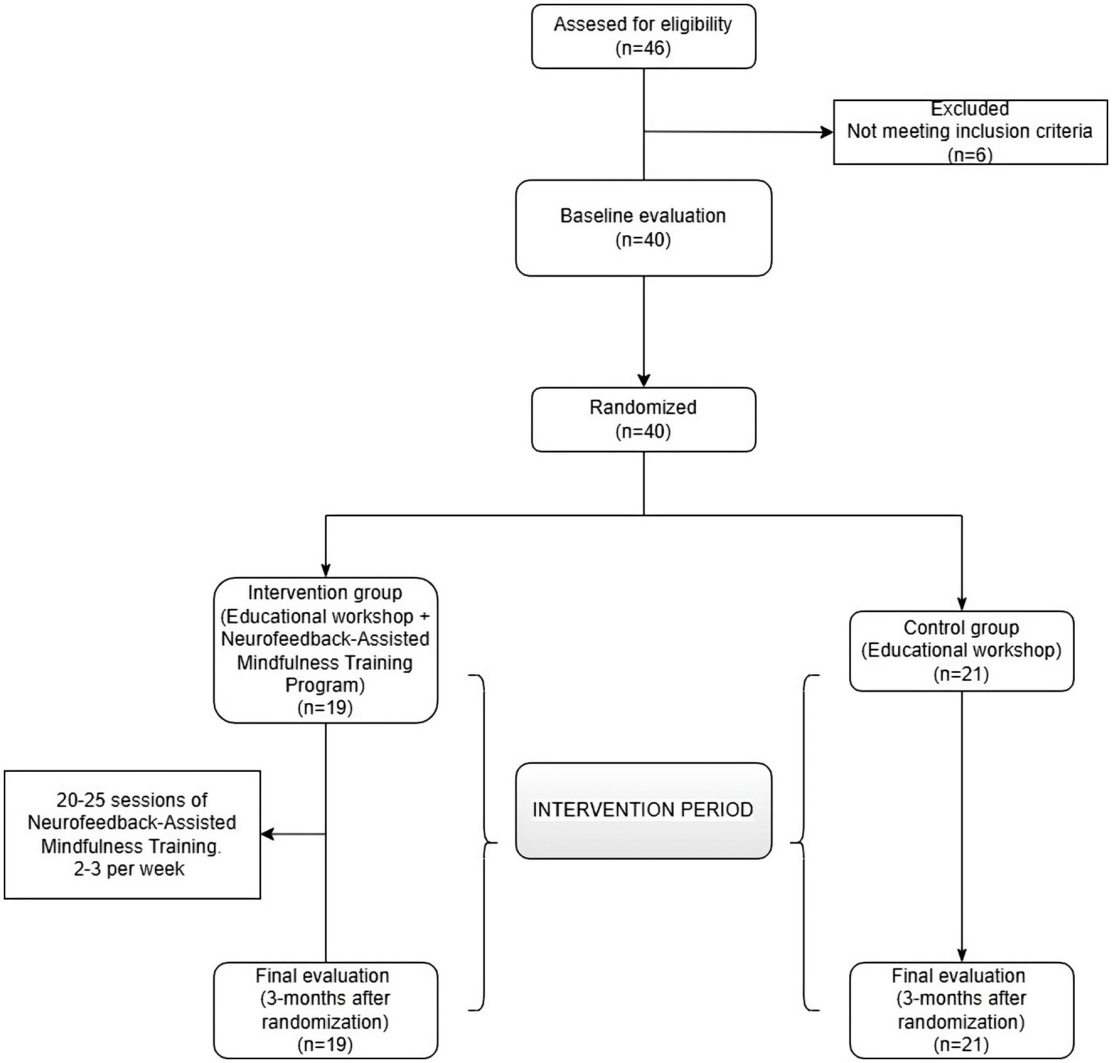
Study flowchart.

The flow of participants through the study is detailed in Figure 1, in accordance with the CONSORT statement.

Initially, a total of 46 participants were assessed for eligibility, of whom 40 met the inclusion criteria and were randomized. Twenty-one participants were allocated to the CG and 19 to the IG. A single participant from the CG was lost to follow-up, as they did not attend the post-intervention assessment despite repeated contact attempts.

Data from this participant were included in the baseline (pre-intervention) analyses used to characterize the study population but were excluded from the post-intervention comparisons (within- and between-group) due to the absence of outcome data.

### 3.2 Characteristics of the study population

The sample consisted of 40 participants (IG: 19 and CG: 21) (Figure 1). The mean age was 24.8 ± 3.4, and 82.5% of the participants were women. Regarding educational level, 64.1% had university studies, and 80.0% were students, of whom half combined their studies with their work activity. Only 20.0% were exclusively dedicated to work. Table 1 shows the specific characteristics of each group.

**Table 1:** Clinical and socio-demographic characteristics.

**Characteristics**	**Intervention group (NF + educational workshop) (*n* = 19)**	**Control group (educational workshop) (*n* =2 1)**	***p*-value**
Age (years)	25.4 ± 3.5	24.2 ± 3.4	0.284
Women (%)	17 (89.5%)	16 (76.2%)	0.270
**Educational level, %**			0.224
Middle or high school	5 (26.3%)	9 (42.9%)	
University studies	14 (73.7%)	12 (57.1%)	
**Work situation, %**			0.496
Student	6 (31.6%)	10 (47.6%)	
Student + employed	8 (42.1%)	8 (38.1%)	
Employed	5 (26.3%)	3 (14.3%)	
Physical activity (METs/min/week)	2,723 ± 1,566	2,853 ± 1,896	0.816
Age of first mobile phone	13.5 ± 3.2	13.6 ± 2.2	0.915
**Main use of the mobile phone (%)**			0.408
Personal	5 (26.3%)	7 (33.3%)	
Recreational	6 (31.6%)	7 (33.3%)	
Personal + professional	–	1 (4.8%)	
Personal + recreational	6 (31.6%)	2 (9.5%)	
All of them	2 (10.5%)	4 (19.0%)	
**Frequency of mobile phone consultation (%)**			0.650
< 15 min	3 (15.8%)	2 (9.5%)	
15–30 min	4 (21.1%)	7 (33.3%)	
30 min−1 h	8 (42.1%)	7 (33.3%)	
1–2 h	2 (10.5%)	2 (9.5%)	
>2 h	–	2 (9.5%)	
Only when notifications are received	2 (10.5%)	1 (4.8%)	
Smartphone Addiction Scale (SAS-SV) score	26.9 ± 9.8	28.0 ± 7.9	0.697
Nomophobia Questionnaire (NMP-Q) score	73.8 ± 28.3	64.6 ± 22.4	0.255
Depression, Anxiety and Stress Scale 21-item (DASS-21) score	19.7 ± 17.1	18.0 ± 10.8	0.443
Athens Insomnia Scale (AIS) score	6.3 ± 3.7	6.8 ± 3.5	0.816

The mean age at first use of the mobile phone was 13.6 ± 2.7 years. The main reasons for use were leisure (32.5%), personal use (30.0%), and both purposes equally (20.0%). With regard to the frequency of use, only 22.5% of participants stated not checking their phone every hour, while the remaining participants consulted it at least once per hour.

### 3.3 Effects of the intervention on anxiety levels and psychological distress

The primary outcome of this study was anxiety, assessed using the anxiety subscale of the DASS-21 scale. In the total sample (CG and IG), anxiety scores decreased by 2.4± 7.7 points at the 3-month follow-up, although this reduction did not reach statistical significance (*p* = 0.057). Significant decreases, however, were observed in the scores of depression and stress subscales after the intervention, as reported in Table 2.

**Table 2:** Changes in the DASS-21 scores in the whole sample.

**Scale / subscale**	**Baseline evaluation (*n* = 40)**	**3-month evaluation (*n* =3 9)**	**Difference**	***p*-value**
DASS-21: anxiety subscale score	9.3 ± 10.6	6.9 ± 7.3	2.4 ± 7.7	0.057
DASS-21: depression subscale score	11.3 ± 9.8	6.7 ± 9.5	4.6 ± 9.1	0.003
DASS-21: stress subscale score	16.8 ± 10.9	12.4 ± 9.0	4.5 ± 8.5	0.002
DASS-21 total score	18.7 ± 14.2	13.0 ± 11.1	5.7 ± 10.3	< 0.001

DASS-21, Depression, Anxiety and Stress Scale 21-item.

**Figure 2:**
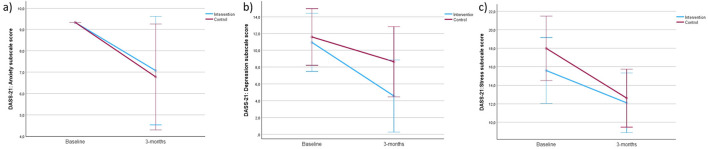
Changes in the DASS-21 subscales in the control and intervention groups from baseline to 3-month follow-up. **(a)** Results in anxiety (*F* = 0.028), **(b)** depression (*F* = 0.998), and **(c)** stress (*F* = 0.576).

Figure 2 shows the changes in DASS-21 subscales in both groups from baseline to 3-months follow-up: (a) Results in anxiety, (b) depression and (c) stress. No statistically significant differences were observed between the IG and the CG in any of the psychological variables assessed. For the DASS scales, the mean differences (IG–CG) were 0.1 (95% CI: −1.6 to 1.9; *p* = 0.868) for anxiety, −2.4 (95% CI: −7.2 to 2.4; *p* = 0.324) for depression, and −1.5 (95% CI: −5.3 to 2.4; *p* = 0.453) for stress. Data on effect size are described in the Figure legend. Both groups showed reductions in each DASS-21 subscale scores at 3 months. This indicates that the experimental intervention was no more effective in reducing anxiety than the control intervention.

### 3.4 Effects of the intervention on mobile phone addiction, nomophobia, and insomnia

At the 3-month evaluation, statistically significant differences were observed in the other study variables compared to the baseline values in the total sample (Table 3). Mobile phone addiction, assessed with the SAS-SV, showed a mean decrease of 4.8 ± 6.8 points. The levels of nomophobia, measured with the NMP-Q, decreased by 10.5 ± 14.4 points. All were highly significant reductions. Finally, insomnia symptoms assessed with the AIS showed a difference of 1.8 ± 2.8 points.

**Table 3:** Changes in secondary study variables in the whole sample.

**Scales**	**Baseline evaluation (*n* = 40)**	**3-month evaluation (*n* =3 9)**	**Difference**	***p*-value**
Smartphone Addiction Scale (SAS-SV) score	27.2 ± 8.7	22.4 ± 6.0	4.8 ± 6.8	< 0.001
Nomophobia Questionnaire (NMP-Q) score	69.7 ± 25.4	59.2 ± 19.0	10.5 ± 14.4	< 0.001
Athens Insomnia Scale (AIS) score	6.7 ± 3.6	5.0 ± 3.5	1.8 ± 2.8	< 0.001

**Figure 3:**
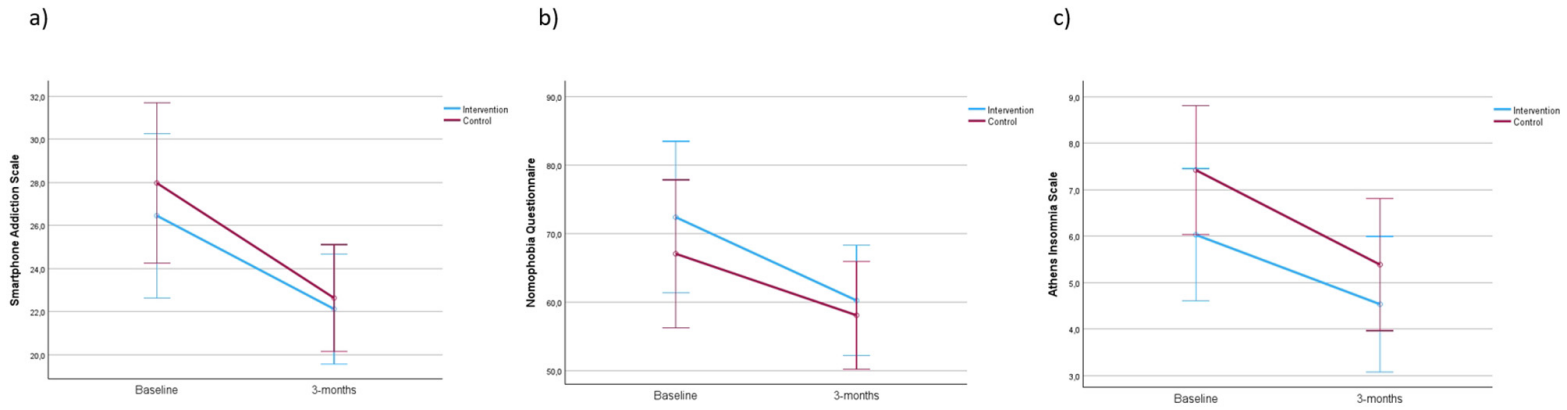
Changes in the smartphone addiction **(a)** (*F* = 0.271), nomophobia **(b)** (*F* = 0.358), and insomnia **(c)** (*F* = 1.619) scales in the control and intervention groups from baseline to 3-month follow-up.

Figure 3 shows the changes in both groups in smartphone addiction (a), nomophobia (b) and insomnia (c), from baseline to 3-months follow-up. Similarly, the mean difference in the SAS-SV score was −1.0 (95% CI: −5.0 to 2.9; *p* = 0.606), while for the NMP-Q score it was 3.7 (95% CI: −8.9 to 16.4; *p* = 0.553). Finally, the AIS showed a mean difference of −1.1 (95% CI: −2.9 to 0.7; *p* = 0.211). Data on effect size are described in the Figure legend. Both groups showed a similar decrease in SAS-SV (a), nomophobia levels, assessed with NMP-Q (b), and insomnia signs (c).

Although both groups showed improvement at 3 months on each variable, there was insufficient evidence to demonstrate that the NAMTP techniques were more effective compared to the educational workshop.

### 3.5 Adherence to the neurofeedback intervention

The IG was composed of 19 participants. Table 4 shows adherence and intervention data. Adherence to the program was 88.8%, with a mean of 22.2 ± 4.1 completed sessions. A correlation analysis has been performed, showing no differences between adherence to the sessions and the outcomes analyzed. No adverse events related to either intervention were reported during the study period. The MUSE^®^ device monitored the time in which the mind remains in a calm state, recording an average of 39.5% of the session time, with an SD of 11.3%. In this group, a mean of 17.3 ± 8.9 recoveries and 13.5 ± 8.9 birds was observed. A correlation analysis has been performed, showing no differences between bird counts and the outcomes analyzed.

**Table 4:** Adherence and intervention data (*n* = 19).

**Variable**	**Mean ±Standard Deviation**
Number of sessions//% adherence	22.2 ± 4.1//88.8%
% Calm during the sessions	39.5 ± 11.3
% Still during the sessions	81.6 ± 13.3
Average heart rate during the sessions	68 ± 8
Number of recoveries	17.3 ± 8.9
Number of birds	13.5 ± 8.9

### 3.6 Self-reported use of technologies and social media

Regarding the time spent using new technologies, the most frequently used devices were the smartphone (mean use of 4.28 ± 2.18 h/day) and the computer (5.37 ± 3.15 h/day). In relation to habitual patterns of use, participants reported spending more time on social media (2.68 ± 2.08 h/day) and chatting or messaging (2.55 ± 1.83 h/day). Although a reduction in screen time was observed in both groups at the 3-month assessment, no statistically significant differences were found between them (*p* > 0.05).

For the IG, significant differences were found in daily smartphone use (*p* = 0.017), time in front of the TV (*p* = 0.048) and time spent chatting (*p* = 0.022), indicating that the intervention effectively influenced participants' behavior in these domains. Statistically significant decreases in computer (*p* = 0.001) and TV (*p* = 0.039) use were also observed for the CG. Both groups showed a significant reduction, at 3 months, in the use of social media, indicating that both the workshop and NF were effective on their own. These results suggest that adding NAMTP enhanced the overall impact on participants' digital habits and routines. However, it cannot be stated that one offered greater effectiveness over the other (Table 5).

**Table 5:** Reduction in the daily use of devices and social media.

**Device use**	**Intervention group (NF** + **educational workshop) (*****n*** = **19)**				**Control group (educational workshop) (*****n*** = **20)**			
	**Baseline evaluation**	**3-months evaluation**	**Difference**	***p*** **-value**	**Baseline evaluation**	**3-months evaluation**	**Difference**	***p*** **-value**
Daily use of smartphone (h/d)	4.63 ± 1.19	3.78 ± 1.07	0.85 ± 1.31	0.017	4.51 ± 2.65	4.11 ± 2.08	0.40 ± 1.54	0.264
Daily use of personal computers (h/d)	5.85 ± 2.42	5.28 ± 2.94	0.58 ± 2.20	0.296	4.60 ± 2.89	3.72 ± 2.72	0.89 ± 1.03	0.001
Daily use of tablet (h/d)	1.76 ± 2.23	1.60 ± 2.38	0.17 ± 0.75	0.611	1.32 ± 0.94	1.89 ± 1.68	−0.57 ± 0.86	0.278
Daily use of televisión (h/d)	0.76 ± 0.88	0.46 ± 0.66	0.30 ± 0.49	0.048	0.89 ± 0.82	0.56 ± 0.69	0.33 ± 0.51	0.039
Daily use of video games (h/d)	0.91 ± 1.65	0.80 ± 1.37	0.11 ± 0.30	0.351	0.31 ± 0.60	0.27 ± 0.50	0.04 ± 0.11	0.356
Daily time in chats or messaging (h/d)	2.81 ± 2.18	2.24 ± 1.83	0.57 ± 1.16	0.022	2.28 ± 1.48	1.94 ± 1.28	0.34 ± 1.08	0.087
Daily time in social media (h/d)	2.56 ± 1.73	1.73 ± 1.10	0.82 ± 1.41	0.010	2.78 ± 2.35	2.03 ± 1.57	0.75 ± 1.78	0.038

No differences between groups were found for any variable.

## 4 Discussion

### 4.1 Principal's findings

The primary objective of this study was to evaluate the effectiveness of a NAMTP in reducing anxiety related to the use of new technologies in young Spanish adults. In this study, NAMTP did not lead to significant improvements in anxiety, mobile phone addiction, nomophobia, or sleep quality compared to CG.

Our findings partially coincide with previous research, such as a clinical trial that found the long-term benefits of NF in improving anxiety and depression may be time limited (61). In the context of internet addiction, a South Korean study found that a mobile NF program improved addiction levels but no significant changes in neurocognitive functions (42). In contrast, research conducted in Georgia reported improvements in different components of attention in children with internet addiction disorders, suggesting that NF could influence key brain regions involved in ADHD and IAD, such as the prefrontal cortex, caudate nucleus, and thalamus (48).

An active CG was selected over a no-treatment or standard care CG for several reasons. Considering the multiple studies highlighting mental and physical issues among young people (12, 15, 62), it was considered ethically inappropriate to withhold a basic preventive measure from the CG. Furthermore, the workshop was designed as a preventive, health-promoting intervention aimed at educating participants on responsible technology use, functioning as a public health measure even for a non-clinical population. The primary goal of the study, however, was to explore the effectiveness of NAMTP rather than to determine its superiority over the workshop. In addition, gold-standard conventional treatment has not yet been established for this emerging field. Finally, providing some form of intervention for all participants was considered beneficial for improving recruitment and retention rates, which often represent a challenge in clinical trials involving volunteer populations.

It should be noted that the studies reviewed use highly heterogeneous NF protocols, varying in the number of sessions, program duration, and the type of technology used (mobile devices vs. clinical equipment). This lack of standardization hinders direct comparison between studies and limits the generalizability of the results. In addition, much of the existing literature originates from Eastern countries, underscoring the need for further research in Western populations, where different cultural and environmental factors may influence the effectiveness of NF.

The current body of NF research presents several methodological limitations that constrain the reliability and generalizability of published findings. Systematic reviews of fMRI-NF and fNIRS-NF studies have identified substantial underpowering, with median statistical power estimates as low as 21–22% for detecting moderate clinical effects. Such underpowering likely contributes to an excess of reported significance, potentially due to selective analysis or reporting bias (63).

Even in laboratory-based mindfulness neurofeedback—where participants can modulate neural targets such as the Default Mode Network (DMN) and frontal midline theta—mechanistic claims remain weak, largely due to the frequent absence of appropriate sham control conditions (64). Outside the laboratory, it also remains unclear what specific brain mechanisms consumer-grade devices target and, consequently, why or how they should alter psychological states. A recent meta-analysis found only modest improvements in psychological distress (Hedges g ≈ −0.16 to −0.29), with no correlation between device-derived EEG metrics (e.g., Muse's “calm” score) and mindfulness or mental health outcomes. This pattern supports the interpretation that the reported benefits may primarily reflect neurosuggestion rather than genuine neural modulation. Users' expectations and the perceived scientific credibility of neurotechnological devices appear to play a substantial role in shaping subjective outcomes (65).

Regarding daily use of the new technologies, one study found that longer screen exposure time is associated with an increased risk of anxiety and depression (66); and that spending more than 3 h per day on social media doubles the risk of mental problems, according to a report by the American Psychological Association (67). In our sample, participants reported between 4 and 6 h daily in front of smartphones and computers, with an average use of social media of 2.68 ± 2.08 h/day, mainly for personal or entertainment purposes. These data coincide with reports from the National Institute of Statistics (INE) of Spain, which places communication, entertainment, and information as the most frequent activities carried out on the Internet among the Spanish population (68).

With regard to sleep, a systematic review found that excessive use of social media is associated with poorer sleep quality, reduced sleep duration, increased difficulty falling asleep, and a higher risk of depression among young people (69). In addition, using screens during the last hour before bedtime has been associated with sleep interruptions (70). In this context, 97.5% of the study participants reported using a mobile phone within 2 h before bedtime, and 57.5% took between 30 min and 2 h to fall asleep. The initial AIS score was higher than 6, indicating the presence of clinically relevant insomnia in part of the sample. Although there was improvement in the AIS score at 3 months in the IG, as observed in other studies, NF has not demonstrated objective clinical efficacy, although subjective improvements have been found (43, 44).

### 4.2 Limitations

This study has several limitations that should be considered when interpreting the results. First, as a single-center study, our results may not be generalizable to other populations. In addition, the sample was predominantly female, with university studies and in an active academic or work situation, which limits the ability to generalize the findings to male populations or others with different sociocultural contexts.

Another significant limitation was that the methodological design did not allow us to completely isolate the specific effect of the NAMTP since both groups received minimal educational intervention, which could dilute the differences between the groups. Also, due to the nature of the intervention, it was not possible to blind either the participants or the researchers. The lack of blinding may have introduced performance bias, as participants' awareness of their group allocation and the expectations of the nurse could have influenced the results.

In addition to the previous limitations, the absence of a “no-treatment” group makes it impossible to determine whether the positive effects observed in both groups were due to the intervention, the workshop, or a placebo effect. Furthermore, the use of a general anxiety scale (DASS-21) rather than a scale specifically designed to measure anxiety related to the use of new technologies may have limited our ability to isolate and accurately measure the specific impact of the intervention.

Finally, the lack of standardization in NF protocols reported in the literature, as well as the paucity of studies in Western contexts, limits direct comparison and applicability of the findings to other populations and clinical settings.

### 4.3 Implications for practice and future research

The findings of this study underscore the need to view problematic technology use not merely as an acute issue but as a chronic condition requiring long-term management within clinical practice. This study suggests that simple psychoeducational support, delivered in primary care and community settings, was considerably effective.

Future research should prioritize methodological rigor through adequately powered randomized controlled trials (*n* ≈ 100 for medium effects), employing consistent control conditions and standardized NF protocols. However, enhancing methodological consistency alone may not be sufficient. Integrating psychoeducational components and social scaffolds—such as guided reflection, peer support, and instructor-led elements—could improve adherence and facilitate a deeper understanding of mindfulness principles.

If the broader aim is to promote a rational and health-conscious use of these technologies, the most impactful future direction may lie in public health education. The goal is not to discourage their use, but to encourage users to engage with them in a mindful, critical, and responsible way. Educational initiatives that raise awareness about both the potential benefits and limitations of these technologies could help ensure they are used to support wellbeing rather than contribute to excessive or unreflective use.

## 5 Conclusions

The NAMTP did not demonstrate significant improvements in reducing anxiety, mobile phone addiction, or nomophobia. No significant effects were observed on sleep quality either. These findings suggest that, although NAMTP may be a feasible and well-tolerated intervention, further research with larger samples and consistent control conditions is needed to determine its potential benefits for anxiety reduction and behavioral regulation related to technology use.

## Data Availability

The original contributions presented in the study are included in the article/supplementary material, further inquiries can be directed to the corresponding author.
